# The prevalence of polycystic ovary syndrome in a community sample of Iranian population: Iranian PCOS prevalence study

**DOI:** 10.1186/1477-7827-9-39

**Published:** 2011-03-25

**Authors:** Fahimeh Ramezani Tehrani, Masoumeh Simbar, Maryam Tohidi, Farhad Hosseinpanah, Fereidoun Azizi

**Affiliations:** 1 Reproductive Endocrinology Research Center, Research Institute for Endocrine Sciences, Shahid Beheshti University of Medical Sciences, Tehran, Iran; 2 Obesity Research Center, Research Institute for Endocrine Sciences, Shahid Beheshti University of Medical Sciences, Tehran, Iran

## Abstract

**Background:**

Despite the heavy burden and impact of the polycystic ovary syndrome (PCOS) in reproduction and public health, estimates regarding its prevalence at community levels are limited. We aimed to ascertain prevalence of PCOS in a community based sample using the National Institute of Health (NIH), the Rotterdam consensus (Rott.) and the Androgen Excess Society (AES) criteria.

**Methods:**

Using the stratified, multistage probability cluster sampling method, 1126 women were randomly selected from among reproductive aged women of different geographic regions of Iran. PCOS were diagnosed using universal assessment of ultrasonographic parameters, hormonal profiles and clinical histories.

**Results:**

The mean +/- SD of age of study population was 34.4 +/- 7.6 years. Estimated prevalence of idiopathic hirsutism was 10.9% (95% CI: 8.9-12.9%); 8.3% of women had only oligo/anovulation and 8.0% had only polycystic ovaries. The prevalence of PCOS was 7.1% (95% CI: 5.4-8.8%) using the NIH definition, 11.7% (95% CI: 9.5-13.7%) by AES criteria and 14.6% (95% CI: 12.3-16.9%) using the Rott definition.

**Conclusions:**

At community level, widespread screening of Rotterdam criteria will increase the estimated prevalence of PCOS over twofold. Establishing an explicit and contemporaneous method for definition and screening of each PCOS criteria has important investigational implications and increase the comparability of published research.

## Background

Polycystic ovary syndrome (PCOS) is the most common gynecological endocrinopathy [1,2]. Women with PCOS are at increased risk of reproductive problems including infertility, endometrial cancer, late menopause [3-6] and also metabolic aberrations, including insulin resistance, type 2 diabetes mellitus, dyslipidemia and cardiovascular diseases [7-10].

Despite the heavy burden and impact of the polycystic ovary syndrome (PCOS) in reproduction and public health, estimates regarding its prevalence are limited; considering the controversy regarding its diagnostic criteria and difficulties in conducting prevalence studies at community levels, data on its current prevalence are questionable [11]. The reported prevalence of PCOS ranges between 2.2% to 26%in various countries, depending on the recruitment process of the study population, the criteria used for its definition and the method used to define each criterion [1,11-21]. Recruitment strategy affects the types of subjects enrolled in a study; e.g. recruiting the subjects using the promise of a health evaluation [19]may potentially bias the results toward disease-carrying individuals. Considering that the Rott. versus NIH criteria increases the PCOS prevalence by 1.5-2 times [21,22], its prevalence may be influenced by using various definitions and the screening method for identification of androgen excess or ovulatory dysfunction [11,14,23]; unless universal screening for hyperandrogenemia or subclinical oligo/anovulation is conducted, identification of these conditions can easily be overlooked [24,25]. There are significant ethnic and racial variations in the clinical presentation of PCOS [26,27]and the Ferriman-Gallwey (F-G) score of hirsutism may be unreliable for identifying androgen excess in some ethnic groups [28]. East Asians are typically less hairy than Euro-Americans, which may be explained by low levels of 5a-reductase activity in the skin of those women [29].

To the best of our knowledge there is no population based study that estimates the prevalence and clinical characteristics of PCOS in the Eastern Mediterranean Region. The objective of the present study was to determine the prevalence of PCOS under the NIH, Rott. and the AES criteria, in a well-defined, non-selected population of Iranian reproductive aged women, using universal assessment of ultrasonographic parameters, hormonal profiles and clinical histories.

## Methods

### Study subjects and the sampling method

Sample size was calculated based on these parameters: P = 0.085 [5], α = 0.95, d = 0.025, cluster design effect = 2 and a non response rate = 0.15. A stratified, multistage probability cluster sampling method, with a probability in proportion to size procedure, was used. The frame for the selection of the sampling units was based on the Iranian household lists available in the Health Department. The information regarding the age, sex, and marital status of each family member is available in this list and updated annually. Selecting the cluster was made systematically and the starting points for the survey in each cluster were determined centrally.

A total of 1126 women, aged 18-45 years, were recruited from among reproductive aged women living in urban areas of four randomly selected provinces of different geographic regions i.e. Ghazvin (Central), Kermanshah (East), Golestan (North) and Hormozgan (South). The age and sex distribution of the population of these provinces is representative of national general population based on 2006 national population and housing census of Iran. Menopausal women, those who had undergone hysterectomy or bilateral oophorectomy and pregnant women were excluded (n = 90). To minimize the effect of treatment bias, all other women, regardless of hormonal therapy including insulin sensitizers and oral contraceptive pills were included, but their hormonal and biochemical parameters were not considered for statistical analysis.

### Study protocol

Pairs of trained staff members of local medical universities/schools (midwives) served as interviewers, and a trained supervisor monitored the process in each district. The interviewers, thoroughly explained the purpose and procedure of the study to subjects and obtained their consent and a checklist questionnaire, based on the inclusion and exclusion criteria, was completed at subjects' homes (n = 1126). Women who met the inclusion criteria (n = 1036) were invited to a referral clinic in each province for a comprehensive interview and physical exam. Ninety seven eligible women who signed the inform consent did not came to the clinics, as a result the response rate of our study was 91%. For those eligible women who referred to clinics (n = 939) a standard questionnaire including demographic and reproductive variables, with emphasis on regularity of menstrual cycle, gynecological history, hyperandrogenic symptoms, family history of irregular menstrual cycle and hirsutism was completed, during face-to-face interviews by trained midwives under supervision of a gynecologist. The hirsutism scores were assessed using the modified Ferriman-Gallwey (mFG) scoring method [30]. Acne was scored based on its number, type, and distribution [31].The menstrual cycle of subjects on hormonal medication was evaluated by questioning about menstrual regularity before starting medication. To maximize the accuracy of hirsutism scoring, subjects with an initial mF-G score more than 3 per the study midwives and/or those women with menstrual dysfunction were reexamined by a single gynecologist in each province.

All participants underwent clinical examinations, where body weight, height, waist (WC), hip circumferences (HC) and blood pressure were measured. Body mass index was calculated as weight in kilograms divided by the height in meters squared (kg/m^2^).

An overnight fasting venous blood sample was obtained from each subject on the second or third day of their spontaneous or progesterone induced menstrual cycles (n = 929). All sera were stored at -80°C until the time of measurements.

All of the study subjects were invited for transvaginal (n = 760) or transabdominal (n = 169) ultrasound scans of the ovaries, which were performed using the 3.5-MHz transabdominal and 5-MHz transvaginal transducer by an experienced sonographer in each province and all scans were assessed by a single sonographer. Ultrasound was performed as the same day as the blood samples were collected.

### Measurements

Dehydroepiandrosterone sulfate (DHEAS), 17-hydroxyprogesterone (17OH-P), Total testosterone (TT) and Androstendion(A4) were measured by enzyme immunoassay (EIA), (Diagnostic biochem canada Co. Ontario, Canada). Sex Hormone Binding Globulin (SHBG) was measured by immunoenzymometric assay (IEMA), (Mercodia, Uppsala, Sweden). All ELISA tests were performed using Sunrise ELISA reader (Tecan Co. Salzburg, Austria).

Luteinizing hormone (LH), Follicle stimulating hormone (FSH), Prolactin (PRL), and Thyroid stimulating hormone (TSH) was measured by immunoradimetric assay (IRMA), (Izotop, Budapest, Hungary) using gamma counter Wallac Wizard, Turku, Finland).

It has been shown that in women the free androgen index (FAI) has a good correlation with free testosterone measured by physical separation method[32]; therefore FAI was calculated using the formula [TT (nmol/L) × 100/SHBG (nmol/L)]. The intra- and inter-assay coefficients of variation for TT were 5.6% and 6.6%; for DHEAS: 2.0% and 5.1%; for 17 OH-P: 4.8% and 6.8%; for SHBG: 1.2% and 5.7%; for A4: 2.2% and 3.5%; for LH- 3% and 5.8%; for FSH: 3.5% and 4%; for TSH: 1.7% 3.4%, and for PRL, they were 2.1% and 4.1%.

### Definitions

We defined PCOS in our study using the NIH[33], Rott.[34] and AES criteria[35]. Using the NIH criteria, PCOS was defined as the combination of chronic anovulation (ANOVU) and clinical hyperandrogenism and/or hyperandrogenemia (HA). By Rott. criteria, PCOS was defined by the presence of two or more of the following: 1) Oligo/anovulation (ANOVU), 2) Hyperandrogenemia and/or hyperandrogenism (HA), and 3) Polycystic ovaries (PCO). Using the AES definition, PCOS was diagnosed by the presence of clinical and/or biochemical hyperandrogenism (HA) with ovarian dysfunction defined as oligo/anovulation (ANOVU) and/or polycystic ovaries (PCO). Hyperprolactinemia, thyroid dysfunction, and nonclassic 21-hydroxylase deficiency were excluded in all of the women who achieved the other criteria for the diagnosis of PCOS.

ANOVU was considered as vaginal bleeding episodes at no less than 35-day intervals [36,37]. HA was determined as clinical hyperandrogenism (CH) and/or biochemical hyperandrogenemia (BH). CH was defined by the presence of hirsutism (mF-G ≥8)[30], acne, or the presence of androgenic alopecia. BH was detected by FAI and/or DHEAS and/or A4 level, above the upper 95th percentile for the 362 women studied, who were not on any hormonal medication and had no clinical evidence of hyperandrogenism, ANOVU and PCO. Specifically, the upper normal limits were total T = 0.88 ng/ml, A4 = 2.3 ng/ml, DHEAS = 246 μg/dL and FAI = 5.47

PCO was diagnosed by the presence of 12 or more follicles in each ovary, measuring 2-9 mm in diameter and/or increased ovarian volume (10 cm3) [38,39].

Idiopathic hirsutism(IH) was defined as hirsutism without ANOVU and/or PCO[24]. BH plus hirsutism was defined as hirsutism with BH without PCOS, using the Rott.definition [40].

Primary infertility was defined as the having the history of trying to conceive for at least one year without success despite of regular sexual intercourse, no use of contraception and no previous pregnancy.

### Statistical analysis

Continuous variables were checked for normality using the one-sample Kolmogorov-Smirnoff test; they are expressed as mean ± standard deviation and/or median and interquartile ranges, as appropriate. The categorical variables are expressed as percentages. Distributions between groups are compared using the Kruskal-Wallis test, followed with Mann-Whitney test with Bonferroni correction for pair wise comparison. The categorical variables are compared using the Pearson's χ^2 ^test. Data analysis was performed using the SPSS 15.0 PC package (SPSS Inc., Chicago, IL).

The ethical review board of the Research Institute for Endocrine Sciences has approved the study proposal and informed consent was obtained from all subjects.

### Details of Ethics Approval

The ethical review board of the Research Institute for Endocrine Sciences has approved the study proposal (approved number: 47854 Date: 15/12/2007) and informed consent was obtained from all subjects.

## Results

**Figure 1:**
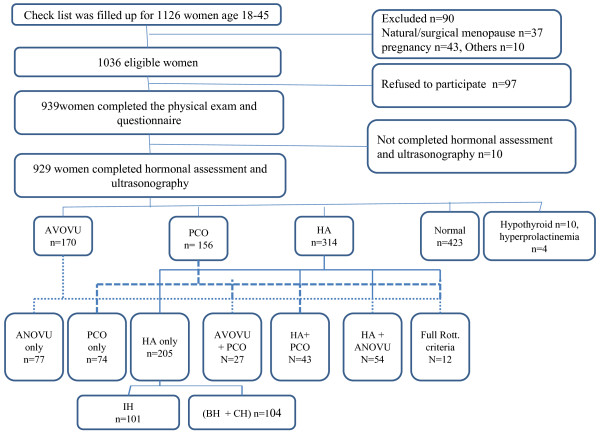
**An overview of study cohort**. ANOVU, oligo/anovulation; HA, biochemical hyperandrogenemia and/or clinical hyperandrogenism; PCO, polycystic ovaries; IH, idiopathic hirsutism; CH, clinical hyperandrogenism; BH, biochemical hyperandrogenemia; Full Rott. criteria Women with all of these three criteria: ANOVU, HA, and PCO.

A study checklist was completed for 1126 women, aged 18-45 years. Figure 1 outlines the data collection procedure. Of 1036 women who met our inclusion criteria, 929 ones completed the study procedure. The mean age of study population was 34.4 years. There was 65 women who used oral contraceptive pill, 61 did so solely for the purpose of contraception.

Of a total of 929 women, 423(45.5%) subjects were eumenorrheic and did not have HA and or PCO (normal), 205 (22.1%) women had only HA and overall, 3.5% of all women had acne. Seventy-seven (8.3%) women had only ANOVU and 74 (8.0%) women had only PCO in ultrasonography; the estimated prevalence of IH was 10.9% (95% CI: 8.9-12.9%) (Figure 1).Out of 929 study participants, 136 women met Rott criteria therefore the prevalence of PCOS using Rott. criteria was 14.6% (95% CI: 12.3- 16.9%). There was 109 women with PCOS based on AES criteria, as a result the prevalence of PCOS was 11.7% (95% CI: 9.5- 13.7%) using AES definition. Out of 929 study participants, 66 women met NIH criteria therefore the prevalence of PCOS using NIH criteria was 7.1% (95% CI: 5.4 -8.8%). There were not any suspicious cases of congenital adrenal hyperplasia, androgen-secreting tumors and Cushing's syndrome based on physical exam and hormonal assessment.

Basic, reproductive and metabolic characteristics of those who had various phenotypes of PCOS using Rott. criteria in comparison to normal women are shown in Table 1. Compared to their normal counterparts, the prevalence of primary infertility was about two to three times higher among women with PCOS. The familial history of androgen excess or ANOVU was more prevalent in group 1 (ANOVU+ HA +/-PCO) phenotype in comparison to normal subjects (Table 1).

**Table1:** The characteristics of various phenotypes of PCOS using Rotterdam criteria

Phenotype characteristic	Group 1 (n = 66) ANOVU+ HA ± PCO	Group2 (n = 43) HA + PCO	Group3 (n = 27) ANOVU + PCO	Normal (n = 423)
**Age (years)**	31.0 (25.8-37.3)*	30.0 (25.0-40.0)*	35.0 (28.0-39.0)	36.0 (30.0-41.0)
**BMI (kg/m2)**	27.0 (25.8-37.3)	25.0 (21.6-29.2)	25.6 (23.0-31.2)	26.4 (23.1-29.4)
**WHR**	0.81 (0.76-0.85)	0.78 (0.75-0.85)	0.83 (0.78-0.86)	0.81 (0.76-0.85)
**Systolic Blood Pressure (mm/Hg)**	110.0 (100.0-120.0)	100.0 (105.0-110.0)	110.0 (100.0-110.0)	110.0 (100.0-120.0)
**Diastolic Blood Pressure (mm/Hg)**	70.0 (60.0-80.0)	70.0 (60.0-70.0)	70.0 (60.0-70.0)	70.0 (60.0-80.0)
**% Women with history of Primarily infertility**	27.3*	21.6*	31.0*	10.6
**% women with family history of HA**	42.4*	20.9	11.1	14.0
**% women with family history of ANOVU**	36.4*†‡	14.0	22.2	19.0
**LH/FSH Ratio**	0.77 (0.46-1.04)	0.64 (0.45-1.0)	0.69 (0.5-1.1)	0.6 (0.43-0.86)
**Total T (ng/ml)**	0.75 (0.57-0.88) *‡	0.81 (0.52-0.93) *††	0.41 (0.29-0.53)	0.51 (0.32-0.68)
**FAI**	4.7 (3.1-6.9) *‡	4.9 (3.6-7.1) *††	2.2 (1.3-2.8)	2.6 (1.5-3.7)
**A4 (ng/ml)**	5.8 (5.1-6.9) *‡	2.1 (1.8-2.5) *††	1.1 (0.9-2.0)	1.1 (0.9-1.7)
**DHEAS (μg/Dl)**	196.1 (149.5-238.1) *‡	200.5 (138.9-248.0) *††	141.5 (45.7-186.7)	138.0 (58.3-192.8)
**SHBG (nmol/L)**	54.8 (42.7-70.7) *	54.4 (45.0- 67.1) *	62.6 (49.1-88.1)	67.2 (53.4-92.4)

Values are given as median (Inter quartile range), *+*The hormonal and biochemical assessments of those women who use insulin sensitizers and/or oral contraceptive pills were not included for statistical analysis. * = versus normal group, p < 0.008, † = Group1 versus Group2, P < 0.008; ‡ = Group 1 versus Group 3; † † = Group2 versus Group3, p < 0.008; WHR, waist to hip ratio; Normal group, eumenorrheic and without HA and PCO; ANOVU, oligo/anovulation; HA, hyperandrogenemia and/or hyperandrogenism; PCO,polycystic ovaries; DHEAS, Dehydroepiandrosterone sulfate; Total T, Total testosterone; A4, Androstendion; SHBG, Sex Hormone Binding Globulin; FAI, Free androgen index.

From among 314 (33.8%) women who had HA in our study, 109 had PCOS using Rott. definition (Figure 1).Of these PCOS women, 54 ones had only CH, 18 women had both CH and BH and the reminding 37 had pure BH (14 pure FAI excess, 6 pure A_4 _excess, 2 pure DHEAS excess and 15 mixed androgen excess). Of these 37, 8 women would possibly have remained undiagnosed, had we not assessed the serum concentrations of androgens in women without oligo/anovulation.

## Discussion

The prevalence of PCOS was 7.1% (95% CI: 5.4 -8.8%) using the NIH definition, 11.7% (95% CI: 9.5- 13.7%) by AES criteria and 14.6% (95% CI: 12.3- 16.9%) using the Rott. criteria in our sample of an Iranian population. To the best of our knowledge there is no study in the Eastern Mediterranean Region that estimates the prevalence and clinical characteristics of PCOS among an unselected population.

The reported prevalence of PCOS in various geographic regions ranges between 2.2% to 26% [1,11-21]. In Southern China the prevalence was 2.4% among 915 women recruited through the offer of a free medical examination using Rott criteria [14]; it was 6.5% among 154 white blood donor women in Spain using NIH criteria [18], In the study by Azziz et al. of women undergoing a preemployment physical examinations in the United States, the cumulative prevalence of PCOS was 6.6% using the NIH definition[1]; the prevalence of PCOS using Rott. definition was reported to be 17.8% among 978 women, who were recruited in a retrospective birth cohort study in South Australia[11]. Among 157 women with type 2 diabetes in Esfahan-Iran, the prevalence of PCOS was 8.2% [41].

The PCOS prevalence depends on the recruitment process of the study population and criteria used for its definition; in the present study considering the Rott. versus NIH criteria increased its prevalence by 2 times, as reported before [21,22]^, ^[42-44]. Furthermore the definition of each criteria and its screening method has a considerable impact on PCOS prevalence at the community level [1,11,16]. There was not any agreement regarding the cut off value for F-G score as a criterion for clinical hyperandrogenism or the menstrual cycles' intervals as a criterion for ANOVU, furthermore there are no agreed definition of hyperandrogenism. In our study, clinical hyperandrogenism was determined as mF-G ≥8 and ANOVU was defined as vaginal bleeding episodes at no less than 35-day intervals; some of the other investigators used different cut of points for these definitions [11,14]; e.g. the clinical hyperandrogenism was defined as mF-G ≥6 by Knochenhauer et al.[16] and as mF-G ≥7 DeUgarte et al. [28]. We did not measured the mid luteal phase serum progesterone and it has been shown that 14-40% of eumenorrheic women with androgen excess and 3.7% of eumenorrheic, non hirsute women have oligoovulatory cycles diagnosed by serum concentration of progesterone [24,25,45]. Therefore, the number of ANOVU women in our study might have been increased from 170 to 222 [(205 × 25%) + (423 × 3.7%)] had we assessed mid luteal phase serum progesterone. To verify hyperandrogenemia, we measured serum concentration of all types of androgens among all our participants, regardless of regularity of their menstrual cycles; if serum concentration of androgen was assessed only among women with ANOVU, or if we did not measure all types of androgens, 8 out of 37 women with only BH would have remain undiagnosed. It seems that the widespread screening of PCOS criteria has a considerable impact on its estimated prevalence at the community level. Recognition of these mild phenotypes of PCOS is a challenging issue and its effectiveness must be evaluated from several perspectives, including the researcher's, clinician's, and the patient's. Azziz et al suggested widespread screening for earlier diagnosis and possibly prevention of serious consequences [46]. However this needs to be confirmed through further well design studies.

The reported prevalence of idiopathic hirsutism varies from 5-29% [19,47]. In the present study, 22.4% of women had only hirsutism, half of them having presented with IH; these women cannot be entirely excluded from the diagnosis of PCOS, because they may have been oligoovulatory, despite of their reported regular episodes of vaginal bleeding [48].

The main strength of the present study is its methodology, as it is a community based prevalence study carried out on an ethnically homogenous population and had an appropriate response rate of 91%. The majority of previous studies on PCOS have relied upon a convenience sample of applicants for university employment [1,16], blood donors [18] or individuals recruited through publicity campaigns [19]. Similar to those reported by Janghorbani et al following a large national survey [49], the educational status and the prevalence of obesity in the present study could justify and confirm our population as being representative of Iranian reproductive aged women. This study also has the advantage of assessment of androgens in all participants, regardless of their menstrual pattern; hence it was unlikely that any hyperandrogenemic subjects were overlooked. The amount of intra-assay variability in our data is also likely to be minimal because all the laboratory measurements were done at the same laboratory by the same person.

Our study does have some limitations; we did not use the menstrual diary to identify menstrual intervals and we did not measure progesterone to identify eumenorrheic women, who had subclinical menstrual dysfunction; our results may therefore be underestimates; for some cases, socio-cultural constraints precluded a vaginal approach for ultrasonography and as a result a specific and sensitive tool was not used for determining the polycystic ovaries. We did not have any information about those few women who refuse to participate in our study, which may also have affected our estimates.

## Conclusions

This study shows that widespread screening of Rotterdam criteria, at the community level, will increase the estimated prevalence of PCOS over twofold. Establishing a clear and contemporaneous method for screening and recognition of PCOS criteria is essential for improving the comparability and potentially the value of published research.

## Competing interests

The authors declare that they have no competing interests.

## Authors' contributions

FRT contributes in study design, execution, analysis, manuscript drafting and critical discussion.MS contributes in study design, manuscript drafting and critical discussion. MT contributes in study design, laboratory testing and manuscript drafting. FH contributes in analysis, manuscript drafting and critical discussion. FA contributes in study design, execution and manuscript drafting. All authors read and approved the final manuscript.
